# Aging, Bone Marrow and Next-Generation Sequencing (NGS): Recent Advances and Future Perspectives

**DOI:** 10.3390/ijms222212225

**Published:** 2021-11-12

**Authors:** Payal Ganguly, Bradley Toghill, Shelly Pathak

**Affiliations:** 1 Leeds Institute of Rheumatic and Musculoskeletal Medicine, University of Leeds, Leeds LS9 7TF, UK; P.Ganguly@leeds.ac.uk; 2 Novogene Europe, 25 Science Park, Milton, Cambridge CB4 0FW, UK; Bradley.toghill@novogene-europe.com

**Keywords:** aging, bone marrow, stem cells, next-generation sequencing (NGS), genomics, inflammaging

## Abstract

The aging of bone marrow (BM) remains a very imperative and alluring subject, with an ever-increasing interest among fellow scientists. A considerable amount of progress has been made in this field with the established ‘hallmarks of aging’ and continued efforts to investigate the age-related changes observed within the BM. Inflammaging is considered as a low-grade state of inflammation associated with aging, and whilst the possible mechanisms by which aging occurs are now largely understood, the processes leading to the underlying changes within aged BM remain elusive. The ability to identify these changes and detect such alterations at the genetic level are key to broadening the knowledgebase of aging BM. Next-generation sequencing (NGS) is an important molecular-level application presenting the ability to not only determine genomic base changes but provide transcriptional profiling (RNA-seq), as well as a high-throughput analysis of DNA–protein interactions (ChIP-seq). Utilising NGS to explore the genetic alterations occurring over the aging process within alterative cell types facilitates the comprehension of the molecular and cellular changes influencing the dynamics of aging BM. Thus, this review prospects the current landscape of BM aging and explores how NGS technology is currently being applied within this ever-expanding field of research.

## 1. Introduction

Aging is a complex process impacting the lives of many people in their 60s and above by means of reduced mobility, increased vulnerability to diseases and poor quality of life (QOL). Biologically, this results from a wide range of cellular and molecular changes over a prolonged period of time [1]. The global population of individuals over the age of 65 was 524 million in 2010, and the number is projected to be 1.5 billion by 2050 [2]. Considering that advancing age is often associated with several diseases [3], the World Health Organization (WHO) predicts that the economic and social burdens associated with these diseases are rising sharply with older age [2] and, thus, are often referred to as age-related diseases (ARDs).

As a field with immense growing interest, the last few decades have seen a rise in exploring the theories and mechanisms that lead to age-related changes and ARDs. Lopez-Otin et al. set the benchmark with their ‘*The hallmarks of aging*’ article discussing the underlying factors and mechanisms that lead to changes in the elderly [4]. Damage to the DNA or the DNA damage responses (DDR) are among the oldest mechanisms accepted for age-related changes. This refers to the accumulation of damage to the DNA or mitochondrial DNA (mtDNA) due to various factors over time that ultimately leads to alterations in the chromosomes and overall genetic instability [5]. Another mechanism observed frequently in aging tissues is the presence of reactive oxygen species (ROS), also known as the free radical theory [6], that have an unpaired electron in their outermost shell, making them unstable. In both healthy and young individuals, antioxidants inherently present within the body balance the presence of ROS. However, with advancing age, the balance is reduced, causing the oxidation of nucleic acids and DNA damage [4]. The shortening of the telomere length (or telomere attrition) with every cell division is an additionally observed mechanism contributing to age-related changes [7]. Furthermore, stem cell exhaustion or the changes deciphered in the number and functions of stem cells also contribute to age-related changes and ARDs. Stem cell exhaustion has also been linked to the other mechanisms of aging and is potentially caused by the previous mechanisms mentioned above [8]. Whilst proteostasis (or protein homeostasis) serves as the quality control for maintaining the proteome, this function is known to decline with age [8]. The loss of proteostasis leads to the presence and accumulation of misfolded proteins that have been linked to ARDS [4,9], making it another hallmark of aging. Finally, changes in the DNA methylation patterns, post-translational histone modifications and chromatin remodelling are epigenetic alterations that can additionally be held accountable as mechanisms contributing to age-relate changes [10].

More recently, aging has been identified as a state of ‘chronic, low-grade, sterile inflammation’ with poorly understood mechanisms. This state is known as ‘inflammaging’ and has been associated with multiple ARDs that contribute to morbidity and mortality in the elderly [11]. Theoretically, inflammaging is an extension of the ‘theories of aging’ and essentially originates from the hypothesis that the complex process of aging and deterioration of health in ARDs must have a common origin [11]. Considering that most ARDs, if not all of them, share an inflammatory pathogenesis, inflammaging is a significant risk factor to the health of the elderly [12].

The lack of ability to fight off infections, cognitive impairment, frailty and immunosenescence are some of the most debilitating impacts of advancing age, causing significant challenges to the QOL within the elderly. Amongst these challenges faced by the elderly is reduced mobility due to alterations in the musculoskeletal system, including symptoms of pain in the joints, inflammation and increased vulnerability towards disorders like osteoarthritis and osteoporosis [13]. Loss of muscle or sarcopenia, decreasing muscle strength, increased stiffness, pain in joints, thinning of cartilage and loss of bone density are widely recognised signs associated with age-related changes and ARDs in the musculoskeletal system [14].

The BM is a critical part of the musculoskeletal system that is known for its dynamic nature in the human body. It is the origin of blood formation or haematopoiesis, serves as the reserve for the different stem cells, bone-forming cells, their progenitors, macrophages and adipocytes, alongside various growth factors, cytokines and other soluble factors [15]. Changes with advancing age in the BM include altered cellularity, increased adipogenesis, myeloid skewing and decline in proliferation and regeneration potential [15]. These changes have frequently been associated with diseases of higher risk like cancer, osteoarthritis and osteoporosis [13]. Evidence suggests that there are similarities between haematopoietic alterations during inflammatory conditions (like infections) and during aging. These include dominant myelopoiesis in relation to immune senescence and impaired B-lymphopoiesis. This is accompanied by increased adipogenesis in the BM and the release of proinflammatory cytokines, including IL-6, TNF-α and IL-1α, and C-reactive proteins. All of these observations provide evidence for the connections linking age-related changes and inflammaging [16].

Considering that BM aging and the resulting changes may have life-altering effects, numerous techniques have been utilised to understand these age-related changes at the cellular level. The investigating parameters, including senescence, proliferation, telomere length, gene expression and reactive oxygen species (ROS), have all aided in the understanding we have today regarding BM aging, including the probable causes behind these mechanisms [4,17]. However, a technique that can provide us with a large amount of data at the genetic level for the identification of new pathways would be ideal for evaluating age-related changes in the BM. Next-generation sequencing (NGS) provides us with exactly that, making it among the most desirable techniques for studying changes with advancing age [18,19].

**Figure 1:**
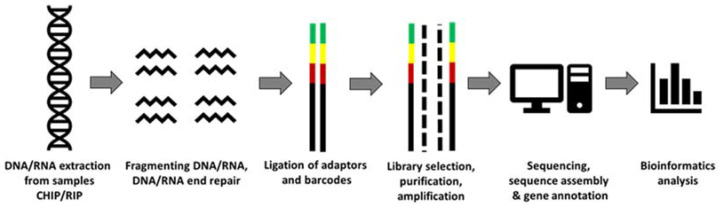
Basic next-generation sequencing (NGS) workflow adapted from Reference [25].

NGS has emerged from the initial sequencing of genomes performed using traditional Sanger sequencing and is thus referred to as second-generation sequencing technology [18]. Fundamentally, the process involves randomly fragmenting DNA/RNA into smaller sizes (typically around 100–300 bp, depending on sequencing strategy), adaptor ligation and immobilisation of the given fragments to construct the DNA or cDNA libraries [20,21]. The latter are lastly amplified to generate multiple copies of each fragment and then subjected to high-coverage sequencing using fluorescence or chemiluminescent-based methods (i.e., bridge amplification) [22]. The resulting reads are finally mapped to the given reference genome of the species, and using assorted bioinformatics pipelines and computational processes, the DNA–RNA, RNA–protein and DNA–protein networks can be evaluated, providing an unparalleled ability to explore various molecular networks [18]. This is briefly outlined in Figure 1. Whilst Illumina, short read and sequencing-by-synthesis remains the ‘current gold standards of clinical and research sequencing’, the scope of NGS has massively broadened within the past five years [23]. The emergence of Pacific Biosciences (PacBio) and Oxford Nanopore Technologies (ONT) long-read sequencing technology (permitting 15 kb-30-kb uninterrupted reads) is a significant contrast from the shorter read lengths provided by Illumina (i.e., 2 × 250 bp on Novaseq 6000™) [24]. Thus, in the context of BM aging, both types of these highly accurate NGS platforms enhance our comprehension of the mutational and evolutionary developments through the understanding of further intricate forms of genetic variations [23].

This review aims at outlining the challenges of the BM with advancing age and how NGS plays a crucial role in helping comprehend these alterations more effectively. We critically review the literature that has been presented until now on BM aging, the application of NGS on BM and discuss the challenges of the field and future perspectives. This review is the first of its kind to focus on BM aging through the lens of NGS.

## 2. Aging in the Bone Marrow

**Figure 2:**
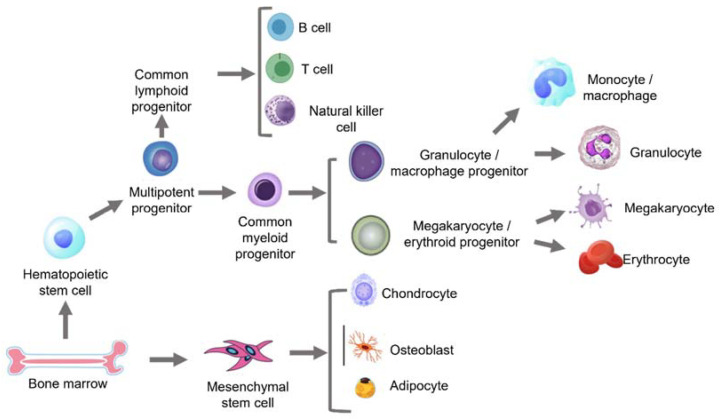
An overview of the lineages arising from the HSC and MSC populations.

The BM serves as the integration between the skeleton and the marrow, providing a niche microenvironment for the cells and growth factors residing here. The BM is home to haematopoietic stem cells (HSCs), mesenchymal stem cells (MSCs) and lymphoid and myeloid progenitors, as well as endothelial cells [26]. Stem cells give rise to different lineages (Figure 2) and are known for their ability to regenerate. However, this ability declines with advancing age, which is now known as stem cell exhaustion, identified as one of the key hallmarks of aging [4,17]. Whilst the different stem cells and immune cells all coexist within the BM niche, how their functions are altered with advancing age have been investigated separately [19]. This section summarises the changes associated with aging amongst the various cell types and the BM in general.

The bone marrow gives rise to both self-renewing HSCs and MSCs. HSCs further turn into multipotent progenitors that form common lymphoid progenitors (CLPs) and common myeloid progenitors (CMPs). CLPs finally form B cells, T cells and natural killer (NK) cells, while CMPs give rise to myeloid cell types that include the different types of granulocytes/macrophage progenitors and megakaryocyte/erythroid progenitors. MSCs differentiate into chondrocytes, osteoblasts and adipocytes, which eventually form the cartilage, bone and fat in the human body, as summarised in Figure 2, which has been recreated and adapted from Wang et al. [27].

### 2.1. The Aging Bone Marrow (BM) Niche

**Figure 3:**
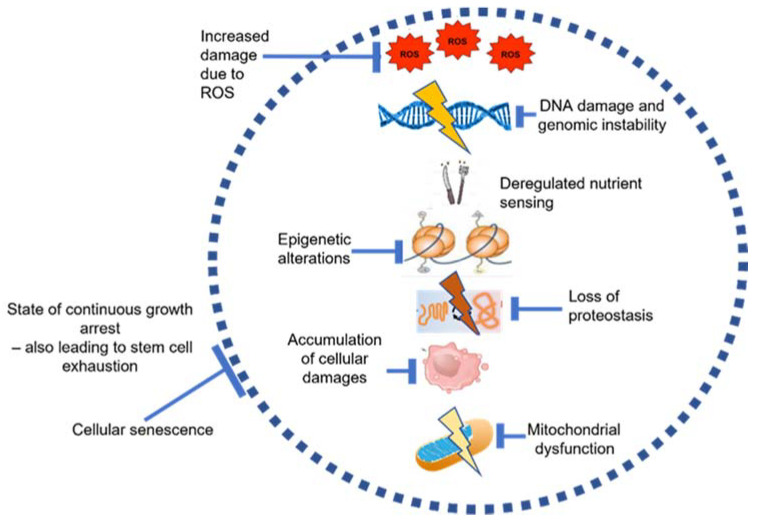
Underlying causes of aging inside the BM, recreated and adapted using information from Lopez Otin et al. [4] and González-Gualda et al. [31].

The BM niche is the unique microenvironment that is provided by the distinctive architecture of the bone, the cellular components and the growth factors and proteins within it. The two major types of stem cells, i.e., haematopoietic stem cells (HSCs) and mesenchymal stem cells (MSCs), give rise to different lineages and, thus, form an essential part of this niche [28]. Being heterogenous in nature, the BM undergoes several alterations with advancing age, including changes in cellularity of the stem cells [29] and loss of the ‘bone–fat balance’ within the BM [30]. Age-related bone loss and decline in bone strength and density, followed by an increased vulnerability to BM and/or bone-related diseases like osteoarthritis, myelodysplastic syndrome and haematological malignancies, are also well-documented [16]. The underlying causative factors for these changes have been proposed to be a combination of several of the ‘hallmarks of aging’, including oxidative stress due to ROS, DNA damage, mitochondrial dysfunction and cellular senescence (Figure 3) [4,17].

More recently, Guidi et al. established that the aged BM niche ‘restrains rejuvenated HSC’. They found that using rejuvenated old HSCs upon transplanting into the BM of young mice was able to partly sustain youthful functions and suggested that age-related changes in stem cells potentially result from both cell intrinsic factors and extrinsic factors (niche) of the BM [32]. Metabolically, an accumulation of free fatty acids (FFA) combined with the decline in the levels of nucleic acids and the amino acid pool has been documented, potentially due to the signature oxidative stress revealed in their transcriptome data associated with mitochondrial dysfunction [33]. Apart from these, the process of myeloid skewing, a process where the lymphoid cells become more myeloid-oriented in the elderly, is also a key indicator of BM aging [34].

Whether it is the altered microenvironment (cell extrinsic factors) leading to the reduced functionality of the cells within the BM with advancing age or the intrinsic properties of the cells or a combination of both these factors influencing this age-related change, investigating changes in the BM cells are key to elucidating the mechanisms of these age-related changes. The fact that the stem cells residing within the BM are self-renewing, being used for various cell therapy applications and are also the progenitors of cells of different lineages and functions makes them the ideal candidates for these investigations and for exploring age-related changes at the single-cell and genetic levels.

### 2.2. Age-Related Changes in Haematopoietic Stem Cells (HSCs)

HSCs were the first identified stem cells and, to this date, remain the stem cells that have been explored extensively for their self-renewal abilities throughout the entire lifetime of an individual. However, they lose their ability to regenerate and replenish the other cell types rising from them with advancing age [35]. While studies have indicated that their numbers increase in the elderly, it has been debated that this increase is a compensatory mechanism for their loss of functionality in the BM. Reports have also suggested that larger fractions of HSCs in the aged are less quiescent and undergo cell division as compared to those in the young, potentially due to the accumulation of higher levels of ROS over time [36]. This, in turn, affects the repopulating capacity of HSCs in the elderly. Strzelecka and Damm discussed in depth the changes observed in aging haematopoietic cells, outlining that the readout of the transcriptome is a good indicator of the cellular state [37].

Along with an increase in the number of cells with decreased functionality, the regenerative and homing capacities of aged HSCs are also shown to be lower in the BM. They suffer metabolic changes [38], impaired autophagy and disrupted protein homeostasis, which altogether impact the regeneration potential of BM HSCs [39]. Furthermore, the myeloid bias is enhanced during both inflammation, as well as during advancing age, leading to myeloid skewing in HSCs, indicating further responses towards the concept of inflammaging [16]. In young individuals, haematopoiesis is normally supported via various HSC clones with similar potential. However, in the elderly, in spite of an overall increase in the number of HSCs, only a smaller proportion of the clones contribute to blood formation [40]. This condition is known as clonal haematopoiesis of indeterminate potential or CHIP, which has increasingly been observed in people between the ages of 55 and 60 and has been linked with haematopoietic malignancies [41].

BM adipocytes cover about 50–70% of the inside of the BM volume in adults and are known to increase with conditions like obesity and diabetes, as well as aging. BM adipocytes have been shown to interact with HSCs and bone cells locally via the secretion of factors like the stem cells factor (SCF), leptin and adiponectin. This potentially impacts HSCs in the BM [42]. While haematopoiesis has been reported to be inhibited by the increase of adipocyte formation in bone, thus altering the rate of formation of HSCs [43], more recently, it has also been reported that BM adipocyte-derived SCF mediates the metabolic regulation of the process of haematopoiesis [44]. This suggests that BM adipocyte-derived SCF and, thus, BM adipocytes are essential to haematopoiesis but may inhibit the process if there is an increase if adipocyte formation within the BM.

### 2.3. Age-Related Changes in Mesenchymal Stem Cells (MSCs)

MSCs (also referred to as skeletal stem cells or SSCs) within the BM are the cells that can form into at least three types of tissues—the bone, fat and cartilage (property often referred to as trilineage differentiation). They are also known for their trophic factors and immunomodulating properties, making them very desirable for extensive use in cell therapy applications [45]. These cells are usually identified in vitro by plastic adherence; trilineage differentiation and by the expression of CD73, CD105 and CD90 as per the International Society of Cell Therapy (ISCT) [46]. However, other markers like SSEA4 and phenotypes CD45^low^ and CD271^+^ are also used for the identification of uncultured BM MSCs [47].

These cells are well-known for regenerative properties, which are reported to decline with advancing age. Studies have reported a decline in their number [48,49], decline in their proliferative potential [50,51], increased bias towards adipogenesis at the expense of osteoblast formation, resulting in decline in bone formation, and enhanced fat formation [52] and decrease in migratory properties [53]. More recently, potential links have been made between the intrinsic type 1 interferon profile within MSCs and their ability to respond to DNA damage [54]. The work by Josephson et al. established the effect of age-related inflammation in MSCs by demonstrating a decline in the number of MSCs and impaired bone regeneration capacity in mice models. They also found that ‘circulating systemic factors’ in the BM of the aged contributed to MSC aging, confirmed by the presence of higher levels of TNF-α, IFN-γ and IL-6 [51], which are also classical indicators of inflammaging.

The probable cause behind these age-related changes in MSCs can also be credited to the hallmarks of aging, including DNA damage, the impact of ROS, telomere attrition and cellular senescence [4]. Even though most of these age-related changes in all the cell types within the BM were initially attributed to cell-intrinsic characteristics, more recently, there has been an increase in acknowledging the effects of the aging BM niche or microenvironment on the exhaustion of stem cells [39]. These usually include the secretion of inflammatory cytokines, presence of a senescence-associated secretory phenotype (SASP) and proinflammatory factors that lead to the formation of a microenvironment that no longer supports the functioning of the cells within it [39].

### 2.4. Age-Related Changes in B Cells and T Cells: Immune Aging and Immunosenescence

The immune system plays a significant role in maintaining the immunity and providing us with protection against infections. The evidence in the literature indicates a decline in the immunological parameters, which has now been termed as immunosenescence [55]. Immunosenescence is shown to cause an accumulation of proinflammatory factors and inflammaging as a result of alterations in the immune cells. The features of immunosenescence include a reduced ability to respond to new antigens, a decrease in the peripheral blood naïve cells and an increase in the frequency of memory cells. These elements, along with inflammaging, are considered to be the hallmarks of immunosenescence [56]. Taking the aforementioned into account, at the cellular level, cells residing within the innate and adaptive divisions become senescent with age. Whilst the exact mechanism pertaining to this phenomenon remains elusive, it is suspected that this may be the consequence of ‘sustained activation in the absence of a specific challenge’ [57].

#### 2.4.1. B Cells

B and T cells function differently during the aging process. Their ability to counteract incoming foreign threats is significantly reduced, thus increasing the risk of infection (56,57). The number of B cells decrease with increasing age, in turn causing a reduced production of high-affinity antibodies. Although this could be attributed to insufficient T-cell assistance, the T-cell-independent responses are also decreased [57]. Notably, Keren et al. demonstrated that the removal of ‘older’ B cells reversed the senescence seen within B cells through the occurrence of lymphopoiesis in the BM [58]. This research not only formed the foundation with which ‘reverse aging’ experiments are based on but reemphasised senescence as a marker of inflammaging. Further exploration of the changes in B cells with advancing age will contribute further to our understanding of aging BM.

#### 2.4.2. T Cells

One of the most prominent features of immunosenescence is the alterations in the T-cell compartment. Naïve T cells are abundant in youth; however, their numbers have been shown to reduce with advancing age. Their lower numbers are often attributed to the process of thymic involution, which also leads to an increase in T-memory cells [59]. Within the T-cell subsets, CD8^+^ circulating T cells are known to be impacted more than other subpopulations with advancing age and are deemed to be the most prominent and consistent markers of immune aging [59]. Recent evidence suggests that changes in CD4^+^ T cells contribute to age-related chronic inflammation and can cause sufficient aging phenotypes in an entire organism [60]. Mittelbrunn and Kroemer also outlined the primary hallmarks of T-cell aging to be defined by the parameters of thymic involution, mitochondrial dysfunction, genetic and epigenetic alterations and, finally, the loss of proteostasis [60].

## 3. NGS and Its Applications in Aging Studies

Previously, there have been studies that explored NGS for a better understanding of age-related changes. It has been used for genome-wide analyses of the networks involved in changes in the retina [61] and human-induced pluripotent stem cell (iPSCS)-derived neurons [62], as well as in HSCs in the BM [63]. As discussed in the former sections, the infamous landmark ‘hallmarks of aging’ paper initially denoted the landmarks of aging, with one of these being the accumulation of DNA damage [17]. In resonance with this and commonly seen with aging is the clonal expansion of mutated stem cells within the BM, leading to an increased risk of haematological malignancy development [64,65]. For example, each decade, an average of 1.3 ± 0.2 somatic mutations are developed per HSC, and age-related haematopoietic clones in individuals over the age of 55 is a relatively common finding [66]. Whilst most of these mutations may not render any functional effects, it has been observed that some of these genome aberrations render cells independent of specific external growth factors or develop a resistance to certain external growth factors, thus facilitating uncontrolled clonal expansion [67]. In the context of BM aging, NGS has thus paved the way to allow scientists to probe the genome, exome and transcriptomic profiles of cells residing within the BM in an attempt to understand the underlying molecular mechanisms.

### 3.1. Targeted Sequencing Approaches

#### 3.1.1. DNA Panels

Aberrant stem cell functions are not just limited to aging but possibly impact autoinflammatory, immunodeficiency and haematopoietic malignancies. Such single-cell transcriptomic approaches, targeted cell sequencing and/or targeted gene panel DNA sequencing can be employed to further study the genetic factors contributing to, or even causing, the disease itself and/or its progression. Single-nucleotide polymorphisms (SNPs) and variations [68] and copy number variations are those parameters most examined when investigating DNA [69].

We recently demonstrated that clonal haematopoiesis was not associated with Schnitzler Syndrome (SchS) patients—an autoinflammatory disease postulated to be associated with a restricted pool of clones within the BM. We studied a panel of 28 genes associated with the development of clonal haematopoiesis and myelodysplastic syndrome, another BM disorder. Solely, one SchS patient demonstrated a nonsense mutation in *STAG2* with a low variant allele fraction, and independently, this could represent a CHIP mutation [70].

#### 3.1.2. ScRNA and Bulk RNA-Seq

Single-cell RNA sequencing (scRNA-seq) has evolved into an effective tool for the identification and classification of cellular populations through examining the transcriptional signature. In the context of the aging BM and associated conditions, several studies have been performed to characterise cellular subtypes and unearth cell-intrinsic differences. We describe two key examples of such studies below.

In 2018, Hennrich et al. sorted HSC populations into six subsets: CD34^+^, lymphocytes, monocytes/macrophages, granulocytic precursors, erythroid precursors and MSCs, using flow cytometry [71]. In accordance with their protein studies, both single-cell RNA-seq on the CD34^+^ subset, as well as bulk RNA sequencing on the sorted populations, revealed that the mRNA transcripts of age-increased glycolytic enzymes were markedly higher in the myeloid compartments as compared to the lymphoid counterpart. This sequencing was conducted using the Switch Mechanism at the 5’ end of the RNA templates (SMART-seq); a single-cell protocol developed to enable a complete genome coverage, thereby permitting the detection of alternative transcript isoforms and single-nucleotide polymorphisms (SNPs) [72]. With the multi-experimental approach, the aforementioned study highlighted that aging triggers changes within the BM niche, specifically reducing the pathway functions involved in human haematopoietic stem and progenitor cell homing [71].

Parallel to the work by Hennrich et al. [71], one group of researchers set out to create a set of reference data for a subset of cells with the healthy human BM to ‘facilitate and validate the analysis of large databases of scRNA-seq’ [73]. Twenty healthy individuals between the ages of 24 and 84 years were recruited to donate BM aspirates, which notably is a challenging task with regards to both ethical perspectives and given the invasive procedure [74]. Firstly, the scRNA sequencing was performed on BM mononuclear cells (BMMCs) at a read depth of 50,000 reads per cell, detecting an average of 880 genes per individual cell. Notably, this section of the study employed the widely used and increasingly popular 10X methodology—a droplet-based system permitting the genome-wide expression profiling of thousands of cells at the same time. The quantification of unique molecular identifiers (UMIs) directly corresponds to the levels of gene expression [75]. Application of the single-cell trajectory analysis combined with the statistical method: *t*-distributed stochastic neighbour embedding (t-SNE) permitted the detection of the lymphoid, myeloid and erythroid lineages with their developmental stages, in concordance with the flow cytometry performed on the matched samples [73]. As an additional resource, the authors performed whole-transcriptome sequencing on eight donors as a further prediction of the proportion of cellular subsets. This second body of bulk RNA sequencing work utilised the Tru-seq library preparation method, widely known for the resulting high-quality libraries from lower amounts of starting RNA material [76]. Whilst the initial aim of this research was to provide a reference dataset for future studies to compare their data to the obtained data, their work revealed an increased expression of chromatin markers in both the HSC progenitor cell populations and the differentiated immune cells, consistent with studies using alternative methodologies [77]. This helped in further affirming the validity of the data obtained.

## 4. The Future of NGS in Aging Studies—Potential, Perspectives and Limitations

### 4.1. Potential and Perspectives of NGS in Aging Studies

With all of the above information, we propose the use of NGS for BM aging in progenitor cells and immune cells from human donors of a wide age range (18–90 years old). This will help to understand the changes with ‘healthy aging’ and potentially underpin the early markers of inflammaging in older adults (40–60 years old), considering skeletal aging has been suggested to begin from the fifth decade of human life [78]. Comparative investigations of transcriptome reads from healthy older adults and from donors with ARDs will help in narrowing down the common genes/genetic profiles that are present in healthy older adults but are potentially further exacerbated in ARDs.

We predict that approaching the ever-dynamic BM and its aging cells will not only help us understand the differences in the genome with healthy aging but will also help with the early diagnosis of various age-related conditions that are commonly observed in the BM. These results could aid in slowing the progression of debilitating diseases like cancer and enhance the QOL of patients through more personalised therapies. Nevertheless, despite the rapid development in the field of NGS within the past few years, several challenges remain, both within the workflow and also with the data output and downstream analysis.

### 4.2. Limitations of NGS in Aging Studies

Within the workflow, technical challenges like poor cellularity, the low quality of the extracted genetic materials and the heterogeneity of the tissue sources are often discussed in detail before the experimental design of these experiments. Considering that the number of several cell populations (MSCs [13] and B cells [79]) and functionality of others (HSCs [80] and naïve T cells [81]) decline with advancing age, extracting sufficient quantities of genetic material from samples of older donors will pose challenges during the library preparation for sequencing. Apart from the extraction of a sample for the genetic material, another predominant challenge lies within the vast amount of data generated, raising storage concerns (e.g., one alignment file (BAM file)); accounting for 30× the coverage of the human genome would approximately generate 90–95 Gb of data. Applying this to simply 10 samples would thus generate nearly 1 Tb of data itself. Whilst such files can be converted into smaller, compressed files, the retention of all raw data is largely necessary for reprocessing purposes. Thus, researchers need to account for the computing expertise, power and space required to accommodate the resulting data. In these lines, one would also encourage the sharing and deposition of such NGS data in public domains. The National Center for Biotechnology Information (NCBI) has an ever-growing bank of data deposited by researchers.

## 5. Conclusions

The last two decades have experienced accumulating evidence in the field of aging, inflammaging and ARDs to enhance our understanding in the field and potentially treat the conditions for a better QOL for the elderly. Currently, the presence of TNF-α, IFN-γ and IL-6, followed by change in the number of cells, distorted functions and skewed differentiation outlines the aging BM for us. However, to find new genes and potential targets that may be associated with aging in the BM, a unique technique like NGS will help underpin pathways that are yet to be discovered.

Focussing on gene panels aimed at the proliferation, differentiation, migration and homing of these cells with advancing age (Table 1) will also add to our knowledgebase of aging BM. We also predict that investigations following the hallmarks of ‘DNA damage’, ‘mitochondrial damage’, ‘cellular senescence’, ‘effect of ROS’ and ‘epigenetic alterations in the human transcriptome’ will provide us with further evidence about the existing pathways and novel factors that may lead to potential targets for therapies in geriatric science.

## Figures and Tables

**Table 1:** List of key studies performed using NGS in aging BM investigations.

Cells Explored	DNA/RNA	Key Findings	Reference
SSCs	RNA	RNA-seq data linked the functional loss of SSCs to diminished transcriptomic diversity of SSCs, transforming the BM in aged mice. Aged SSCs promoted osteoclast activity and myeloid skewing in HSCs	[82]
Non-HSCs (MSCs)	RNA	Provided insights into the BM cellular components of non-HSCs and their transcriptional intermediates along differentiation paths Explored non-HSC subpopulations and differentiation hierarchies for maturing stromal cells	[83]
MSC lineage cells	RNA	Confirmed age-related changes established in MSCs (decline in number and differentiation potential) Identified a new subset of marrow adipocyte lineage precursors (MALPs) within the aging BM	[84]
HSCs and MSCs	RNA	Enhanced central carbon metabolism in the elderly indicating higher anabolic activity in them reminiscent of the Warburg effect Decrease in factors responsible for homing, egress and differentiation (CXCL12, VCAM and integrins) decreased in the elderly	[71]
BM cells with focus on T cells	RNA	T-cell population increased with advancing age Provided compatibility between mass cytometry, RNA-seq and flow cytometry across donors aged between 24 and 84 years old	[73]
